# Pandemic Influenza School Closure Policies

**DOI:** 10.3201/eid1302.061109

**Published:** 2007-02

**Authors:** Laura H. Kahn

**Affiliations:** * Princeton University, Princeton, NJ, USA

**To the Editor:** Holmberg et al. (*1*) are rightly concerned that state pandemic plans in the United States represent a patchwork without central coordination or direction. These concerns are particularly relevant for school closure decisions during an influenza pandemic. The US Department of Health and Human Services’ checklist regarding school closures gives conflicting messages (*2*). For example, it recommends that schools stay open during a pandemic and develop school-based surveillance systems for absenteeism of students and sick-leave policies for staff and students. It also recommends developing alternate procedures to ensure the continuity of instruction in the event of district-wide school closures. These vague recommendations may reflect the paucity of data to recommend school closure.

To assess the current status of school closure decisions in the United States, I conducted an internet survey of all 50 state health commissioners during the spring of 2006. I asked the respondents 2 questions: “Who makes the school closure decisions in your state?” and “What absenteeism rate, if any, would prompt a school in your state to close during a typical influenza year and/or during a pandemic influenza year?” Of the 44 responding states, I found that school closure decisions were primarily a local-level responsibility in half. Of these 22 states, closure decisions would be made either on a school-by-school or a school district–by–school district basis. Only 6 states indicated that school closure decisions would be made at the state level, and 16 states would have decisions made jointly at the state and local levels (Table).

**Table:** Number of states reporting influenza pandemic school closure policies at various levels, USA*

Region	Local only	State and local	State only
Northeast	4	2	0
South	7	8	2
Midwest	7	3	0
West	4	3	4
Total†	22	16	6

*Northeast: CT, DC, MA, ME, NH, NJ, NY, PA, RI, VT; South: AL, AR, DE, FL, GA, KY, LA, MD, MS, NC, OK, SC, TN, TX, VA, WV; Midwest: IA, IL, IN, KS, MI, MN, MO, ND, NE, OH, SD, WI; West: AK, AZ, CA, CO, HI, ID, MT, NM, NV, OR, UT, WA, WY. †Six states did not respond.

For a typical influenza season, only 6 states indicated that they close schools if a certain absenteeism rate due to illness were reached. For 5 of these states, the absenteeism rates ranged from 10% to 30%; the sixth state said its schools would close if the rates were anywhere from 7% to 31%. However, only 1 state reported a threshold absenteeism rate for closure during an influenza pandemic. Another state said that it was developing an absenteeism rate that would prompt closure for pandemic influenza. Forty-two states did not have threshold absenteeism rates that would prompt school closures during an influenza pandemic.

In July 2006, the Department for Education and Skills in the United Kingdom published guidelines regarding school closure (D. O’Gorman, pers. comm.). Although the final decision for school closure would lie with local school officials, the national government might advise all schools and childcare facilities to close when a pandemic reached their area to reduce the spread of infection among children (*3*). It is believed that all would comply with closure advice and that use of emergency powers under the UK Civil Contingency Act 2004 to require services to close would not be necessary. If all British schools in an area were advised to close during a pandemic, the situation would be reviewed after a period of time, such as 2 to 3 weeks, by local officials acting on information from the UK government, to decide whether to remain closed.

Although the United States is a nation dedicated to federalism, an uncoordinated approach for community response measures such as school closure decisions could jeopardize our efforts in containing a deadly pandemic. If schools were to remain open until a certain percentage of students and faculty became ill, as they do during typical influenza seasons, then control measures to contain the outbreaks would likely be far more difficult to achieve because a chain of transmission would be established. Some might consider it unethical for schools to stay open in the face of a pandemic with a high death rate. I therefore think a national policy, or at least specific national guidelines, should be developed jointly by the Centers for Disease Control and Prevention and the Department of Education, so that states’ school districts can develop rational, coherent, and coordinated closure plans to protect children and communities during an influenza pandemic.
